# Neutropenic Enterocolitis Disclosing an Underlying Cyclic Neutropenia

**DOI:** 10.1155/2020/8858764

**Published:** 2020-12-01

**Authors:** Fatima Ezzahra Lahlimi, Khawla Khalil, Soumia Lahiaouni, Illias Tazi

**Affiliations:** Hematology Department, University Hospital Mohamed VI, Faculty of Medicine, Cadi Ayyad University, Marrakech, Morocco

## Abstract

Neutropenic enterocolitis is a syndrome characterized by fever and abdominal pain in a neutropenic patient. It is often reported in children treated for leukemia and rarely reported in patients with other diseases. Herein, we report the case of a 9-year-old patient with a medical history of recurrent fever and mouth ulcers since the age of 4, who presented with neutropenic enterocolitis complicated with intestinal perforation which all leaded to disclose cyclic neutropenia. The patient was successfully treated by aggressive supportive care combined with surgical intervention. Neutropenic enterocolitis with possible complications should be considered and promptly managed in every neutropenic patient and may reveal a rare cause of neutropenia as cyclic neutropenia.

## 1. Introduction

Neutropenic enterocolitis (NE), also named as typhlitis, is a critical condition that is associated with high mortality, due to inflammatory necrotization of the cecum, and it may extend to the ileum and ascending colon. It is mainly observed in patients profoundly myelosupressed [1–4].

Cyclic neutropenia (CN) is a rare hematologic disorder that is characterized by repetitive episodes of neutropenia occurring at more or less regular intervals. During neutropenic periods, the commonest infections are pharyngitis, periodontis and cutaneous infections [5–7]. We report the case of cyclic neutropenia disclosed by neutropenic enterocolitis complicated with intestinal perforation. A brief overview of the relevant literature is given, focusing on the diagnosis and the optimal management of this rare life-threatening association.

## 2. Case Presentation

A 9-year-old boy born to nonconsanguineous parents was admitted to the pediatric emergency department with complaints of progressive abdominal pain and fever during the last 24 hours. His medical history revealed recurrent episodes of fever, pharyngitis, and diarrhea since the age of 4. The symptoms had cyclical pattern of 21 days and persisted for about a week. On the initial physical examination, he presented a fever of 39.6°C, blood pressure at 90/60 mmhg, and heart rate at 100 beats per minute. There was an abdominal tenderness and guarding, especially in the right lower quadrant (RLQ). No superficial lymph nodes or hepatosplenomegaly were palpated. He had no pulmonary signs, and his heart examination was normal. The remainder of the examination was unremarkable.

Laboratory investigations on admission showed a total white blood cell count of 3000/mm^3^, an absolute neutrophil count of 340/mm^3^ (1500–8000/mm^3^), a hemoglobin level of 13 g/dl, and a platelet count of 174000/L. The bleeding time and coagulation time were normal. The C-reactive protein was 268 mg/L (<3 mg/L). The lactate dehydrogenase (LDH) was 420 UI/L (200–400).

A computed tomography (CT) scan of the abdomen showed signs of increased bowel wall thickness of the small intestines and ascending colon at 8 mm (normal 4 mm). Bacterial and fungal cultures revealed no growth. Stool polymerase chain reaction assay was negative. Various other laboratory tests were performed. Serology for hepatitis A, hepatitis B, hepatitis C, human immunodeficiency virus, and cytomegalovirus was negative. Results of an Epstein–Barr virus serologic test were consistent with the previous exposure. Serologic tests were negative for antinuclear and antinative DNA antibodies. Bone marrow aspiration examination demonstrated a maturation arrest of granulocytic cells.

The patient was immediately started on ceftriaxone 100 mg/kg/day i.v. and amikacin 15 mg/kg/day i.v. according to the local epidemiology. He was switched to imipenem after 48 hours of persistent fever. He received IV morphine for the pain, fluid resuscitation, and correction of electrolyte imbalance. He was also put on bowel rest.

The patient's fever resolved two days later, and he experienced substantial improvement of abdominal pain. Neutrophil recovery was within 7 days, and repeated imaging after 2 weeks showed resolution of colonic inflammation and he was able to go back home.

**Figure 1:**
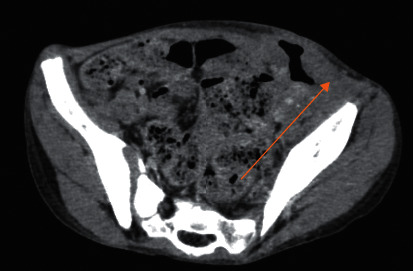
This collection is fistulized against the skin at the RLQ (arrow).

**Figure 2:**
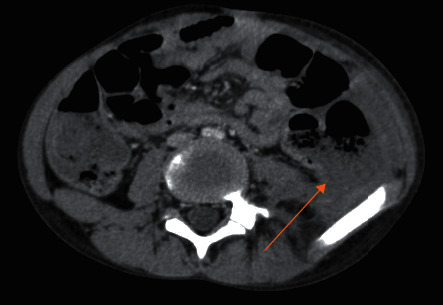
Abdominal CT: solution of continuity of the colonic wall with collection of neighbors (arrow).

Three weeks later, he was admitted to our emergency department for recurrence of fever and severe abdominal pain. He was neutropenic with an absolute neutrophil count at 340/mm^3^, and CT scan showed local necrosis, an intestinal perforation, and enterocutaneous fistulas (Figures 1 and 2). Urgent surgery was indicated. Laparotomy, resection of the involved bowel, and ileostomy were performed. Pathological analysis of the specimen revealed findings of diffuse loss of mucosa, hemorrhage, and a necrotic surface. The patient was immediately started on imipenem and received supportive measures (bowel rest with nasogastric decompression, fluid resuscitation, and supplemental nutrition).

Serial measurements of absolute neutrophil counts indeed demonstrated fluctuating neutrophil counts with a 21-day periodicity and neutropenia at the nadir of the cycle, and a possibly underlying cyclic neutropenia was suspected. Additional tests (including detection of ELANE (elastase, neutrophil expressed) mutation) were not performed.

A granulocyte-colony-stimulating factor (G-CSF) treatment was started. Two days later, the patient's fever subsided. G-CSF therapy was continued at progressive doses till neutrophil recovery. The patient condition improved remarkably, and timely follow-up is being performed in order to manage further neutropenic episodes properly.

## 3. Discussion

Neutropenic enterocolitis (NE), also known as typhlitis, is an inflammatory process with intramural infection of the distal ileum, cecum, and ascending colon that occurs predominantly in neutropenic patients with leukemia. The clinical presentation is characterized with the triad of neutropenia, fever, and abdominal pain [1–4]. Its true occurrence is unknown. Its exact pathogenesis is poorly understood and believed to be multifactorial. The pathophysiological of cyclic neutropenia is incompletely defined. The majority of CN patients harbor ELANE (elastase, neutrophil expressed) mutations, but the precise mechanism of cyclic fluctuations in blood cells and the detailed molecular mechanisms are not understood. Premature apoptosis of myeloid precursors within the marrow is the cause of the reduced neutrophil production. It is due to the production of misfolded elastase proteins and may also induce the unfolded protein response [8, 9].

Neutropenia is the main risk factor as it causes impaired host defense to microbial invasion to the bowel wall. It is mainly described in leukemic patients who have received chemotherapy. It is also encountered in patients severely myelosuppressed due to therapy for solid tumors, autologous bone marrow transplant, and rarely in patients with cyclic neutropenia. Complications include necrosis, hemorrhage, perforation, and septicemia [10, 11].

When it comes to imaging characteristics, it is believed that CT is the imaging option of choice for the rapid diagnosis of NE with findings such as right lower quadrant inflammatory mass and pericecal fluid, inflammatory changes in the pericecal soft tissues, or bowel wall thickness of greater than 4 mm. Abdominal X-ray may show small nonspecific signs such as bowel obstruction, a paucity of gas in the right lower quadrant, or a dilated and fluid-filled cecum. It can also show free intraperitoneal air, a complication of bowel perforation [3, 12].

Our patient presented initially with NE. CT scan showed signs of bowel wall thickness and was treated initially with antibiotics. However, the underlying cause remained a mystery.

Cyclic neutropenia (CN) is a rare hematologic disease characterized by cyclic reduction in the granulocyte proliferative pool in the bone marrow and the release of mature neutrophils. It manifests typically at an approximately 21-day cycle with a range of 14 to 35, lasting 3 to 6 days per episode. During the period when there are few circulating neutrophils, patients commonly suffer from fever, painful mouth ulcers, gingivitis, lymphadenopathy, pharyngitis, tonsillitis, and bacterial infections. Skin infections such as impetigo are also common. However, serious infections and septicemia are rare [5–10].

It is a very challenging diagnosis as the neutropenic phase only lasts for few days per cycle, and clinical manifestations such as recurrent fever and susceptibility to infection may be overlooked for a long time. Our patient spent the last five years with recurrent fever and mouth ulceration, and the diagnosis was only suspected after being admitted in our department.

It is because of the medical history of periodic fever and infection during the last 5 years, the admission for the NE, and the occurrence of fever, intestinal perforation, and neutropenia 21 days later that cyclic neutropenia was disclosed.

Early surgery is indicated in NE to treat complications such as perforation, peritonitis, gangrenous bowel, or severe gastrointestinal bleeding. Primary anastomosis is believed to have a high incidence of complications in neutropenic patients. Functioning ileostomy is recommended because of poor healing and risk of infection. Mortality rates range from 2.2 to 48%, and the prognosis depends on the underlying disease and on the clinical conditions of the patient [13, 14].

## 4. Conclusion

Cyclic neutropenia can be easily overlooked due to its rarity and nonspecific symptoms. It may be the underlying cause to life-threatening conditions such as neutropenic enterocolitis and intestinal perforation.

## Data Availability

No data were used to support this study.

## Abbreviations

NE: Neutropenic enterocolitis
CN: Cyclic neutropenia
ELANE: Elastase, neutrophil expressed
G-CSF: Granulocyte-colony-stimulating factor
LDH: Lactate dehydrogenase
RLQ: Right lower quadrant.
